# Effects of Growth Stage and Particle Size on the Physical, Chemical, Structural, and Bioactive Properties of Alkaline-Extracted Dietary Fiber from Rice Husk (*Oryza sativa* L.)

**DOI:** 10.3390/foods14173094

**Published:** 2025-09-03

**Authors:** Tipaukson Chaikwang, Apichaya Bunyatratchata, Pornpisanu Thammapat, Jiranan Ratseewo, Sirithon Siriamornpun

**Affiliations:** 1 Department of Food Technology and Nutrition, Faculty of Technology, Mahasarakham University, Kantarawichai, Maha Sarakham 44150, Thailand; 67010853502@msu.ac.th (T.C.); apichaya.b@msu.ac.th (A.B.); 2 Research Unit of Thai Food Innovation (TFI), Mahasarakham University, Kantarawichai, Maha Sarakham 44150, Thailand; 3 Technology Program, Faculty of Agricultural Technology, Rajabhat Mahasarakham University, Maha Sarakham 44000, Thailand; thammapat.p@gmail.com; 4 Division of Food Innovation and Technology, Faculty of Liberal Arts and Science, Sisaket Rajabhat University, Sisaket 33000, Thailand

**Keywords:** rice by-product, cellulose, β-glucan, antioxidant capacity, phytosterols

## Abstract

Rice husk (RH), an underutilized by-product of rice milling, represents a promising source of dietary fiber with health-promoting potential. This study investigated the effects of growth stage and particle size on the chemical composition and functional properties of dietary fiber isolated from green rice husk (G-RH) and ripe rice husk (R-RH). Cellulose contents ranged from 62 to 85 g/100 g DW in G-RH and from 38 to 73 g/100 g DW in R-RH, while β-glucan levels were 66–85 mg/g DW and 67–92 mg/g DW, respectively. FTIR and XRD analyses confirmed the presence of cellulose and its crystalline structure. Particle size reduction enhanced water- and oil-holding capacities (up to five-fold and two-fold, respectively) and increased phenolic, flavonoid, and phytosterol contents. G-RH exhibited higher levels of bioactive compounds and a more diverse phytosterol profile compared to R-RH, as verified by GC-MS analysis. In contrast, R-RH showed greater water-holding capacity, whereas G-RH displayed superior oil-holding capacity, underscoring the influence of growth stage on dietary fiber functionality. Overall, these findings highlight rice husk, particularly G-RH, as a promising functional ingredient for food applications.

## 1. Introduction

Rice (*Oryza sativa* L.) is a staple food for more than 3.5 billion people worldwide, especially in Asia, which is both a major producer and consumer [1]. Thailand is one of the world’s leading rice producers, with an annual paddy rice production of approximately 27.2 million tons, yielding around 18 million tons of milled rice [2]. The rice milling process generates substantial by-products, particularly rice husk, the outermost layer of the grain, which is commercially underutilized despite its high biological potential due to its rich content of dietary fiber and bioactive compounds [3,4].

Dietary fiber can be categorized into two primary types: soluble dietary fiber (SDF) and insoluble dietary fiber (IDF). SDF is associated with reducing the risk of cardiovascular diseases and regulating blood glucose levels [5]. A well-known example is β-glucan, a polysaccharide with (1→3) and (1→4)-β-D-glucopyranosyl linkages, which has been widely recognized for its health-promoting effects, including cholesterol reduction, glycemic control, and stimulation of beneficial gut microbiota [2]. In contrast, insoluble dietary fiber (IDF), primarily composed of cellulose, hemicellulose, and lignin, plays a crucial role in digestive health and also influences the textural properties and functional characteristics of food products [6]. Both β-glucan and IDF have significant potential in the prevention and mitigation of non-communicable diseases (NCDs), such as obesity, type 2 diabetes, and inflammatory bowel disease [6]. Non-communicable diseases (NCDs) represent a major and escalating global health challenge, constituting the leading cause of mortality worldwide. It is projected that within the next two decades, NCDs will account for nearly half of the global disease burden in developing countries [7]. Diabetes, one of the most prevalent NCDs, affects a substantial proportion of the global population. Zhou et al. [8] reported that approximately 828 million individuals were living with diabetes worldwide in 2022, with numbers continuing to rise annually. Projections indicate that by 2050, the global diabetic population may reach 1095 million [9].

Rice husk, the outermost layer of the rice grain, accounts for approximately 20% of the weight of the rice husk and consists of 35–40% cellulose, 20–25% lignin, and 15–20% hemicellulose [3]. Its high cellulose content makes rice husk a potential source of insoluble dietary fiber (IDF) with health-promoting properties. However, studies on rice husk are still limited compared to rice bran, for which the effects of extraction methods on the chemical composition and functional properties of rice bran dietary fiber have been extensively investigated [5,10]. Although some studies have reported the extraction of nanocellulose from rice husk using various methods, information on the chemical and functional characteristics of dietary fiber extracted from rice husk remains scarce, particularly regarding the effect of particle size, which is well known to influence both chemical composition and functional properties of dietary fiber. Studies on the effect of particle size reduction on dietary fiber properties have been conducted on rice bran, which has a composition similar to that of rice husk. These studies have demonstrated that reducing particle size can significantly improve dietary fiber properties. For example, Tian et al. [6] reported that reducing the particle size of rice bran during extraction increased the porosity of IDF but decreased its water- and oil-holding capacity, sugar adsorption, and enzyme inhibition. Similarly, Yin et al. [11] found that reducing the particle size of soybean insoluble dietary fiber increased the surface area, thereby enhancing water-holding capacity, swelling, emulsifying ability, and suspension stability. In addition, Zhao et al. [12] reported that superfine IDF powder from rice bran exhibited higher water-holding capacity, swelling capacity, and nitrite ion adsorption capacity compared to conventional IDF, although oil-holding capacity was lower. Particle size reduction also enhanced the extraction of phenolic compounds and antioxidant activity. Moreover, rice husk is a source of bioactive compounds, such as flavonoids (2.1–2.7 mg RE/g DW) and total phenolics (1.1–2.2 mg GAE/g DW), which exhibit strong antioxidant activity, as indicated by DPPH (73–88%) and FRAP (14–18 μmol FeSO_4_/g DW) assays, highlighting its potential as a bioactive ingredient [4]. In addition, studies on the developmental stage of rice grains and rice bran, which are components similar to rice husk, are particularly significant. Lin & Lai [13] reported that the developmental stage of rice grains significantly affects the levels of bioactive compounds in rice bran, including phenolics, flavonoids, tocopherols, tocotrienols, and γ-oryzanol. This finding is consistent with the study by Jiamyangyuen et al. [14], which indicated that the developmental stage of rice bran influences both the content and composition of bioactive compounds. These data are valuable for analyzing the bioactive compounds in rice husk, which represents a key component of the rice grain with varying developmental stages.

The primary objectives of this study were to extract dietary fiber from rice husks harvested at different grain maturity stages and to evaluate the effects of particle size on their physical, chemical, structural, and bioactive properties, including phytosterol content. Particular attention was given to the green rice husk (G-RH), which has not been extensively investigated in previous studies. The research question guiding this work was how the growth stage of rice husk and variations in particle size influence the composition and functional properties of alkali-extracted dietary fiber, and whether these factors are associated with its potential as a functional food ingredient. To address this question, we hypothesized that dietary fiber extracted from green rice husk (G-RH) possesses superior bioactive and functional properties compared to that from ripe rice husk (R-RH), and that particle size reduction further enhances these properties by increasing surface area and accessibility of bioactive compounds. The outcomes of this study are expected to provide fundamental insights into the functional potential of rice husk-derived dietary fiber, identify optimal processing conditions for maximizing its value, and support the sustainable utilization of rice milling by-products. Ultimately, the findings aim to contribute to the development of innovative, functional food ingredients.

## 2. Materials and Methods

### 2.1. Materials and Reagents

Rice husk (RH) from *Oryza sativa* L. at two growth stages, green rice husk (G-RH) and ripe rice husk (R-RH), as described in Table 1, was obtained from Roi Et, Thailand. Chemicals used for β-glucan analysis were purchased from Megazyme International Ireland Ltd. (Wicklow, Ireland). All other chemicals and reagents were obtained from Sigma-Aldrich Co. (St. Louis, MO, USA). Analytical or HPLC-grade (≥90% purity) standards were met for bioactive compounds, antioxidants, and phytosterols.

### 2.2. Sample Preparation

All samples were dried at 60 °C for 4 h. The dried rice husks were then ground using a blender. The ground rice husk without sieving was classified as the “coarse” fraction. The portion that passed through a 50-mesh sieve was defined as the “medium” fraction, while the portion that passed through an 80-mesh sieve was defined as the “fine” fraction. The processed samples were stored in aluminum foil bags and labeled as G-RH and R-RH for rice husks from the green rice husk and ripe rice husk, respectively. The samples were kept at −20 °C until analysis.

### 2.3. Dietary Fiber Extraction

**Figure 1:**
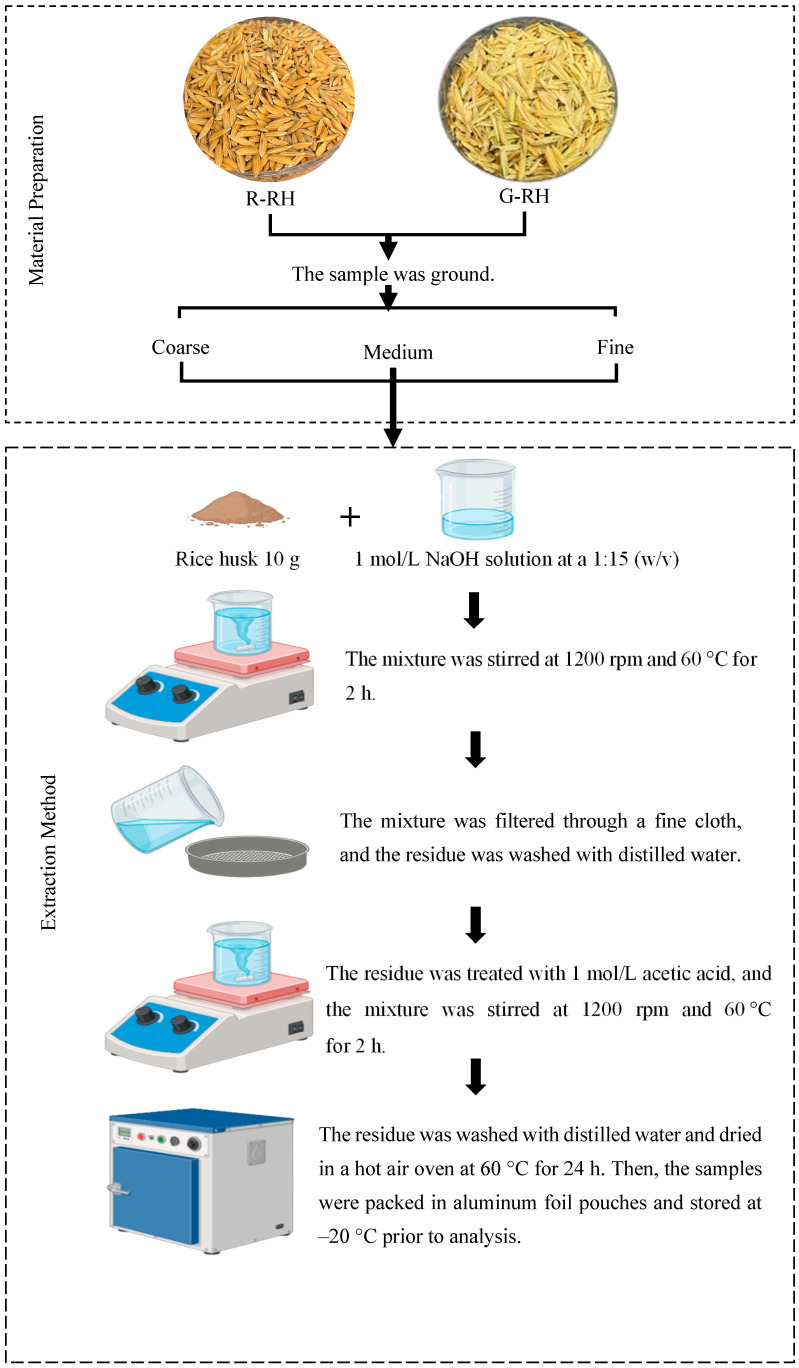
Schematic representation of the process used for extraction of fiber from rice husk. R: fully ripe stage, G: green stage, and RH: rice husk.

The method for extracting dietary fiber from rice husk was adapted from Ullah et al. [15]. Briefly, 10 g of rice husk prepared in 2.2 were weighed and mixed with 1 mol/L NaOH at a ratio of 1:15 *w*/*v*, then stirred at 1200 rpm for 2 h at 50 °C. The mixture was then filtered through a fine cloth, and the sample was washed with distilled water. The residue was subsequently treated with 1 mol/L acetic acid and stirred at 1200 rpm at 60 °C for 2 h. Finally, the sample was dried in a hot air oven at 60 °C for 24 h. After being packed in aluminum foil pouches, the samples were kept at −20 °C prior to analysis. The process of dietary fiber extraction is presented in Figure 1.

### 2.4. Physicochemical Properties Analysis

#### 2.4.1. Color, Moisture Content, and Water Activity (a_w_) Analysis

Color values were measured using a chromameter (Minolta, Model CR-400, Tokyo, Japan), with calibration performed using standard black and white tiles prior to measurement. Moisture content was determined using a moisture analyzer (MX-50, Tokyo, Japan). Water activity was determined using a water activity meter (Pullman, WA, USA).

#### 2.4.2. Water-Holding Capacity (WHC) Analysis

The WHC was determined following the method described by Liu et al. [5]. A 0.5 g portion of the sample was mixed with 20 mL of distilled water and agitated at 540 rpm for 30 min. The mixture was subsequently centrifuged at 3000 rpm for 15 min. The mass of the resulting wet precipitate was recorded, after which it was oven-dried, and the difference in weight was calculated as follows:

WHC (g/g) = (Wet residue − Dry residue)/Dry residue(1)

#### 2.4.3. Oil-Holding Capacity (OHC) Analysis

The OHC was determined according to the method by Ma et al. [10]. A 0.5 g sample was mixed with 10 mL of rice bran oil in a 50 mL centrifuge tube and stirred at 540 rpm for 30 min. The mixture was then centrifuged at 3000 rpm for 15 min. The weight of the precipitate was recorded, and the difference in weight was calculated. Oil adsorption capacity was expressed as the amount of oil (g) absorbed per g of the sample (g/g).

#### 2.4.4. Swelling Capacity (SC) Analysis

The swelling capacity of dietary fiber was determined using a method modified by Liu et al. [5]. A 0.5 g sample of dry dietary fiber was added to a 25 mL graduated cylinder, and the initial volume was recorded. Next, 5 mL of distilled water was added to the sample, which was then soaked and allowed to swell at room temperature for 5 h. The swelling capacity was expressed as the volume of water absorbed per gram of sample (mL/g). The final volume of the swollen dietary fiber was recorded. SC was calculated using the following equation:

SC (mL/g) = swollen volume − original volume/original dry dietary fiber weight(2)

### 2.5. Chemical Composition Analysis

The chemical composition of the sample, including moisture, ash, lipid, and protein, was determined according to the AOAC methods 969.19, 900.02, 920.176, and 920.177, respectively [16]. Cellulose content was analyzed according to the methods of Liu et al. [5]. Results were reported as g per 100 g of dry weight (g/100 g DW).

### 2.6. β-Glucan Content Analysis

The β-glucan content was determined using a cereal β-glucan assay kit (K-BGLU, Megazyme, Wicklow, Ireland) following the manufacturer’s protocol. The analyses were performed in triplicate, and the absorbance was measured at 510 nm using a UV–Vis spectrophotometer (UV-1700, Shimadzu, Tokyo, Japan). The β-glucan content was calculated and reported as mg per 100 g of dried weight (mg/100 g DW).

### 2.7. Structural Analysis

#### 2.7.1. Scanning Electron Microscopy (SEM) Analysis

Micrographs were obtained using a scanning electron microscope (HITACHI TM4000Plus, Ibaraki, Japan). The dietary fiber samples from rice husks were in a dry powder form. Each sample was mounted onto aluminum stubs using double-sided carbon adhesive tape, secured with aluminum tape, and then sputter-coated with gold prior to examination. SEM observations were performed at an accelerating voltage of 10 kV. Micrographs were acquired at magnifications ranging from 150× to 2000× in backscattered electron mode (BSM), with three images captured per magnification for each sample.

#### 2.7.2. Fourier Transform Infrared Spectroscopy (FT-IR) Analysis

FT-IR spectroscopy was employed to identify the functional groups present in the sample using an INVENIO S/Lumos II spectrometer (Bruker, Zurich, Switzerland). Spectral data were recorded in transmission mode within the wavenumber range of 4000–400 cm^−1^ at a resolution of 4 cm^−1^.

#### 2.7.3. X-Ray Diffraction (XRD) Analysis

The crystal structure of the samples was analyzed with an X-ray diffractometer. D8 Advance (Bruker, Karlsruhe, Germany), operated at an accelerating voltage of 40 kV and a current of 40 mA. The diffraction patterns were recorded in the 2θ range of 5° to 40° at a scanning rate of 2°/min, following the method described by Sang et al. [17].

### 2.8. Bioactive Compounds and Antioxidant Activities Analysis

#### 2.8.1. Preparation of Sample Extraction

The extract preparation for total flavonoid content (TFC), total phenol content (TPC), and antioxidant activity analysis was carried out using a modified method adapted from Wanyo et al. [4]. Briefly, 5 g of the sample was extracted with 25 mL of 80% methanol and incubated at 37 °C for 16 h. The resulting mixture was filtered through Whatman No. 1 filter paper, and the filtrate was collected in a brown glass bottle for further analysis.

#### 2.8.2. Total Flavonoid Content (TFC) Analysis

The determination of total flavonoid content was conducted as previously described by Wanyo et al. [4]. A 10 mL test tube was used to prepare the reaction mixture by combining 500 µL of the extract with 2.25 mL of distilled water. Subsequently, 150 µL of 5% NaNO_2_ solution was added, followed by 300 µL of 10% AlCl_3_·6H_2_O solution. The mixture was allowed to react for 6 min before adding 1.0 mL of 1 M NaOH solution and mixing thoroughly. The absorbance was then measured at 510 nm. Flavonoid content was reported as mg of quercetin equivalents per 100 g of dry weight (mg QE/100 g DW).

#### 2.8.3. Total Phenol Content (TPC) Analysis

The procedure was adapted from Boonarsa et al. [18], in which 0.5 mL of 10% Folin–Ciocalteu reagent was mixed with 0.2 mL of the sample extract. This was followed by the addition of 2.25 mL of 7% Na_2_CO_3_ solution, and the mixture was allowed to react at room temperature for 90 min. The absorbance was subsequently measured at 725 nm using a UV–vis spectrophotometer (UV-1700, Shimadzu, Tokyo, Japan). The total phenolic content was determined and reported as milligrams of gallic acid equivalents per gram of dry weight (mg GAE/g DW).

#### 2.8.4. DPPH Free Radical Scavenging Assay Analysis

The DPPH assay was evaluated by a slight modification in the DPPH assay method, as described by Siriamornpun et al. [19]. A 0.06 mM DPPH solution was prepared, and 0.5 mL of the sample solution was mixed with 4.5 mL of the DPPH solution. The mixture was homogenized using a vortex mixer and incubated in the dark for 30 min. The absorbance of the sample was measured at 510 nm. The results were reported as mg of L-ascorbic acid per 100 g of dry weight (mg AA/100 g DW).

#### 2.8.5. Ferric Reducing Antioxidant Power (FRAP) Assay Analysis

Ferric reducing antioxidant power assay mean concentrations of each sample were determined as outlined by Siriamornpun et al. [19], with minor adaptations. Briefly, 0.06 mL of the extract, 0.18 mL of water, and 1.8 mL of FRAP reagent were combined. The FRAP reagent was prepared by mixing 0.3 M acetate buffer (pH 3.6), 10 mM 2,4,6-Tripyridyl-s-triazine (TPTZ) dissolved in 40 mM HCl, and 20 mM FeCl_3_ in methanol in a 10:1:1 volume ratio. The sample was vortexed and maintained at 37 °C for 4 min in a water bath prior to measuring the absorbance at 593 nm. The antioxidant activity was reported as mg FeSO_4_ per g of dry weight (mg FeSO_4_/100 g DW).

#### 2.8.6. Phytosterol Analysis by GC-MS

Phytosterols were quantified following the method described by Siriamornpun et al. [19], with slight modifications. A 2 g sample was used for analysis, starting with matrix hydrolysis via alkaline hydrolysis. The experiment was performed by adding 2 mL of a 600 g/L potassium hydroxide solution, 2 mL of a 10 g/L sodium chloride solution, 2 mL of ethanol 95%, and 5 mL of pyrogallol-ethanol 60 g/L. Subsequently, 1 mL of the internal standard, 5α-cholestane in heptane 1% *w*/*v* (≥97.0% purity), was added. The reaction tube was incubated in a water bath at 70 °C with continuous shaking for 45 min. After incubation, the sample was cooled in an ice bath before adding 15 mL of a 10 g/L sodium chloride solution. Lipid extraction was performed twice using n-hexane/ethyl acetate 9:1 *v*/*v* with a volume of 15 mL. The organic phase was collected and evaporated to dryness using a rotary evaporator at 40 °C under vacuum. The dried residue was then derivatized using 10 mL of N,O-Bis (trimethylsilyl) trifluoroacetamide: Trimethylchlorosilane or BSTFA:TMCS (99:1) and 1 mL of pyridine 99% at 60 °C for 30 min. The resulting derivatives were dissolved in 2 mL of heptane and subsequently subjected to analysis. The analysis was performed using a Thermo Scientific TRACE™ 1610 Mainframe Gas Chromatograph (Waltham, MA, USA). A 1 μL sample was injected via an autosampler in split injection mode at 280 °C. Separation was achieved using a TG-5MS capillary column (30 m × 0.25 mm ID × 0.25 μm) (Waltham, MA, USA) with helium as the carrier gas at a flow rate of 1.0 mL/min. The column temperature program was initiated at 60 °C, held for 1 min, increased at 30 °C/min to 250 °C, held for 1 min, and further increased at 1 °C/min to 280 °C, and held for 13 min. Quantitative determination was achieved by evaluating the ratio of the analyte peak area to that of the internal standard.

### 2.9. Statistical Analysis

All experiments were conducted in three replicates, and the outcomes are presented as the mean ± standard deviation to reflect the variability of the data (*n* = 3). The normality of the data was verified using the Shapiro–Wilk test, which confirmed that the data followed a normal distribution (*p* > 0.05). One-way ANOVA was therefore applied, and Duncan’s multiple range test (*p* < 0.05) was used for mean comparison. Statistical analyses were performed using SPSS version 17 (Chicago, IL, USA) and Origin 2022 version 9.95 (OriginLab Corporation, Northampton, MA, USA).

## 3. Results and Discussion

### 3.1. Physicochemical Properties

#### 3.1.1. Color, Moisture Content, and Water Activity

**Figure 2:**
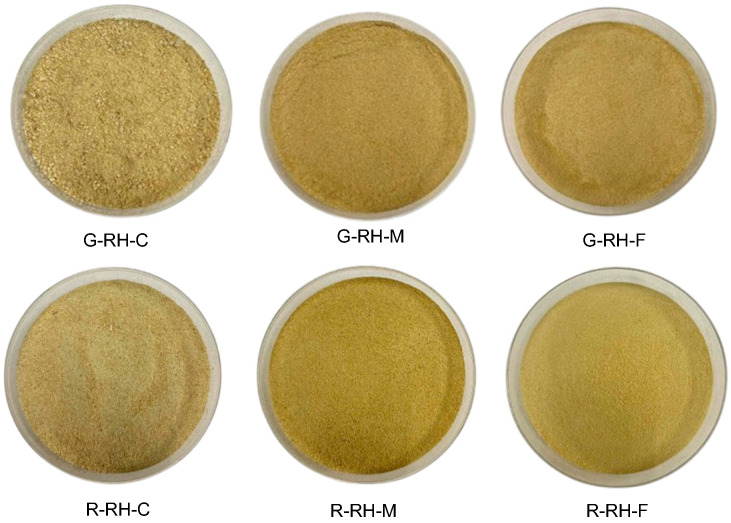
Appearance of dietary fiber obtained from rice husk. R: ripe stage; G: green stage; RH: rice husk. C: coarse; M: medium; F: fine.

Color is a crucial factor that influences the sensory attributes of food products and is closely associated with consumers’ sensory acceptance. [20]. The appearance of dietary fiber obtained from rice husk is shown in Figure 2. Table 2 presents the color parameters of dietary fiber extracted from rice husks at different grain developmental stages. Both the developmental stage and particle size significantly affected the color values. The G-RH and R-RH samples exhibited L* values ranging from 65.19 to 78.62, a* values from −0.73 to 2.07, and b* values from 15.84 to 46.27. The G-RH and R-RH samples exhibited L* values (lightness) ranging from 65.19 to 78.62, indicating that the samples were relatively light in color. The a* values (redness) ranged from 0.73 to 2.07, suggesting a slight tendency toward red, while the b* values (yellowness) ranged from 15.84 to 46.27, reflecting a noticeably yellow hue. Compared to G-RH, R-RH exhibited a higher b* value, indicating that R-RH tended to be brighter and more yellow than G-RH. In contrast, the a* value of R-RH was lower than that of G-RH, suggesting that G-RH had a more intense red tone. Regarding the effect of particle size on color attributes, both G-RH and R-RH showed a decreasing trend in L* values as particle size decreased. This observation aligns with the findings of Bazán-Colque et al. [21], who reported that reducing particle size affected the color characteristics of corn-derived fiber. Similarly, a* and b* values tended to increase as particle size decreased, which is consistent with the results of Liu et al. [22], who demonstrated the influence of particle size on the color properties of dry cornstarch.

In terms of moisture content and water activity (a_w_), both are critical indicators for ensuring microbiological safety in food products [23]. Table 2 shows that G-RH and R-RH samples with coarse particle sizes had the highest moisture contents, followed by those with medium and fine particles. This suggests that smaller particles tend to retain less moisture, possibly due to variations in surface area and moisture exchange mechanisms associated with particle size [5,24]. However, a_w_ values did not differ significantly among the samples and remained within the microbiologically safe range [23]. This study reveals the basic physical properties of rice husk dietary fiber, an agricultural by-product, with potential for food applications.

#### 3.1.2. Water-Holding Capacity (WHC)

The WHC refers to the ability of a material to retain water within its structural matrix, which plays a crucial role in product development. The WHC values of dietary fiber extracted from rice husk at two different maturity stages are presented in Table 3. Both the maturity stage and particle size of the extracted fiber significantly influenced the WHC. Dietary fiber from fully ripe rice husk (R-RH) exhibited a significantly higher WHC compared to that extracted from the green rice husk (G-RH). When compared with previous studies, the WHC of fiber derived from rice husk was higher than that of fibers from citrus fruits (1.65 g/g), grapefruit (2.09 g/g), apple (1.87 g/g), and banana (1.71 g/g) [25]. The effect of particle size during extraction revealed that coarsely ground samples had the lowest WHC, followed by medium-sized particles, while finely ground samples showed the highest WHC. This result is in agreement with the findings of Wang et al. [26], who reported that reducing particle size using sieves with different mesh sizes significantly enhanced WHC due to increased surface area, which facilitates water absorption. Moreover, WHC showed a positive correlation with cellulose content in the samples. Cellulose plays a key role in water retention, a relationship supported by He et al. [27] and further confirmed by the data presented in Table 4. Additionally, alkaline extraction was found to increase the surface roughness of the fibers and expose functional groups such as hydroxyl groups, thereby enhancing water retention capacity through hydrogen bonding [5,28]. This study highlights that the maturity stage and particle size of rice husk significantly influence its water-holding capacity, providing essential insights for the development of health-oriented food products.

#### 3.1.3. Oil-Holding Capacity (OHC)

The OHC refers to the ability of a material to absorb and retain oil within its structural matrix, as shown in Table 3. The maturity stage and particle size of rice husk dietary fiber significantly influenced its OHC values, which ranged from 1.51 to 3.44 g/g. These values are significantly higher than those reported for fibers from apples (0.60–1.45 g/g) and grapefruits (1.20–1.52 g/g), as reported by Yalegama et al. [25]. A clear trend was observed with respect to particle size: finer particles exhibited higher OHC, followed by medium and coarse particles. This could be attributed to the larger surface area of finer particles, which facilitates greater oil absorption [28]. Additionally, the alkaline extraction method used in this study likely disrupted the cellulose structure and increased porosity, both of which are known to enhance oil retention [29]. Therefore, particle size and extraction method significantly influence the oil-holding capacity of rice husk dietary fiber.

#### 3.1.4. Swelling Capacity (SC)

The SC is regarded as a crucial functional property in food systems, as it contributes to the prevention of product shrinkage and influences the viscosity of specific food formulations [24]. As shown in Table 3, both the maturity stage and particle size of rice husk-derived dietary fiber affected its SC. The SC values of fibers extracted from rice husks at the green rice husk and fully ripe rice husk stages ranged from 0.35 to 1.21 mL/g, with fibers from fully ripe rice husk exhibiting higher SC values. As there are no previous reports on the SC values of dietary fiber from rice husks, direct comparisons are limited. Zhao et al. [12] have reported the SC values for dietary fiber from rice bran, and in comparison, the SC of dietary fiber from rice husks was found to be lower. The differences in SC can be explained by the compositional and structural distinctions between rice bran and rice husk. Rice husk primarily consists of insoluble dietary fiber components, such as cellulose [3]. The highly crystalline and compact structure of cellulose limits water penetration, resulting in lower swelling capacity compared to rice bran. The influence of particle size on SC was consistent for both G-RH and R-RH samples, following the trend: fine > medium > coarse. Smaller particle sizes increase the surface area, thereby enhancing water absorption and retention. This finding aligns with the results of Tian et al. [6], who used steam explosion (SE) combined with particle size reduction (80 and 100 mesh) to extract insoluble dietary fiber (IDF) from rice bran and reported improved SC due to increased surface area. Thus, both particle size and extraction method strongly influence the swelling capacity of rice husk fiber.

### 3.2. Chemical Compositions

Cellulose is an insoluble dietary fiber that plays a vital role in promoting health. Its adequate intake has been shown to support overall well-being, including the reduction in blood glucose and serum cholesterol levels [6]. According to Table 4, both the grain developmental stage and particle size of rice husk significantly influenced the cellulose content. The R-RH sample had much more cellulose than G-RH, likely because the husk was more developed at the fully ripe rice husk stage, leading to a greater amount of fibrous parts. When compared to previous research on extracting cellulose from citrus peels, rice husk-derived dietary fiber contained twice the amount of cellulose [30]. Additionally, particle size had a pronounced effect on cellulose extraction efficiency. Finer particles yielded up to twice the amount of cellulose compared to medium and coarse particles, likely due to the increased surface area that facilitates more efficient extraction [31]. As shown in Table 4, which presents the chemical composition of dietary fiber samples, the moisture content ranged from 1.55 to 4.97 g/100 g DW, consistent with values reported for alkali-extracted rice bran fiber [20]. The ash content ranged from 3.54 to 9.15 g/100 g DW, with the highest values observed in the fine particle size group. This may be due to the accumulation of minerals after the removal of other components [32]. The protein and fat contents were in the range of 1.79–5.01 g/100 g DW and 4.95–8.76 g/100 g DW, respectively. In addition to proximate composition, the monosaccharide profile is an important chemical characteristic of polysaccharide-containing materials. Yang et al. [33] reported that rice hull polysaccharides are composed of arabinose (10%), galactose (44.8%), glucose (29.8%), mannose (9.3%), and xylose (6.1%). Similarly, Montipó et al. [34] analyzed the utilization of rice husk and reported monosaccharide contents of glucose (2.30 g/L), xylose (4.91 g/L), galactose (1.10 g/L), and arabinose (1.16 g/L). The differences in reported monosaccharide contents may be attributed to variations in extraction methods. Nevertheless, this compositional profile is consistent with the biochemical nature of dietary fiber and is relevant to its functional properties. Coarse particles tended to yield lower cellulose content compared to medium and fine particles. After cellulose extraction, coarse samples retained higher levels of residual protein and fat, likely due to the dense structure of larger particles, which may entrap protein aggregates [21]. In contrast, medium and fine particles yielded higher cellulose due to their larger surface area, which improved extraction efficiency. Therefore, reducing particle size markedly improved cellulose extraction efficiency, providing insight into optimizing rice husk fiber processing.

### 3.3. β-Glucan

Cereal β-glucan is a type of soluble dietary fiber (SDF) known for its notable nutritional benefits. Studies have shown that it can help lower serum cholesterol, manage blood glucose levels, and decrease the risk of cardiovascular disease [35]. Table 4 presents the β-glucan content in dietary fiber extracted from rice husk. The results revealed that both the grain developmental stage and particle size had significant effects on the β-glucan content of rice husk-derived fiber (*p* ≤ 0.05). Specifically, rice husk collected during the green rice husk stage (G-RH) exhibited significantly higher β-glucan content compared to that collected at the fully ripe rice husk stage (R-RH) across all particle sizes. In particular, the β-glucan content in G-RH samples was approximately four times higher than the values reported by Phuwadolpaisarn [2], who investigated rice husk harvested at the fully ripe rice husk stage. This result supports the hypothesis that rice grains in the G-RH still possess incomplete structural development, and the aleurone layer, which is known to be rich in β-glucan, may remain partially attached to the husk [36], thereby contributing to the elevated β-glucan content observed in G-RH samples. Moreover, particle size significantly influenced the β-glucan content in both developmental stages of rice husk. A clear trend was observed in which finer particle sizes resulted in higher extracted β-glucan content, with the order fine > medium > coarse. This trend is consistent with the findings of Zhao et al. [37], who demonstrated that reducing the particle size of barley enhanced β-glucan extraction efficiency by increasing the surface area, thereby facilitating the release of β-glucan during the extraction process. These findings confirm that green rice husk is a rich source of nutritionally beneficial β-glucan, with particle size influencing its recovery.

### 3.4. Structural Characterization

#### 3.4.1. Microstructure

**Figure 3:**
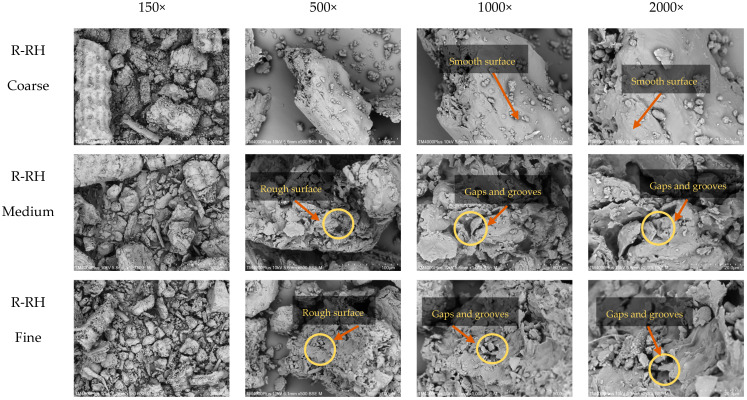

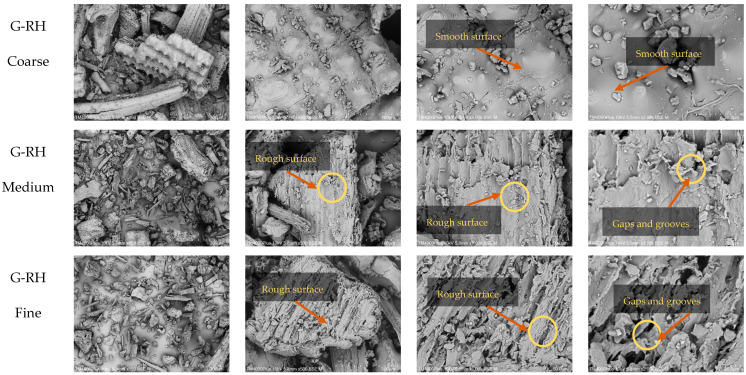
SEM images of rice husk fibers from two different maturity stages at magnifications ranging from 150× to 2000×. Observations were performed at an accelerating voltage of 10 kV. R: ripe stage; G: green stage; RH: rice husk.

The microstructure of dietary fiber extracted from rice husk reflects its distinctive properties and the influence of processing conditions. As illustrated in Figure 3, dietary fiber obtained from coarser rice husk particles displayed a dense, block-like structure with sharp protrusions, likely resulting from the aggregation of non-cellulosic components and other surface-associated substances such as proteins and lipids [6]. These observations may indicate that coarse particle dietary fiber retains higher levels of protein and fat. This observation is consistent with the chemical composition data presented in Table 4, where dietary fibers with coarser particles contain higher protein and fat contents than those with smaller particles. Furthermore, Figure 3 shows that dietary fibers from medium and fine particles have a looser structure, more porosity, and a rougher surface than coarse particles. These changes can be attributed to the increased surface area of smaller particles, which enhanced alkali penetration and facilitated the removal of non-cellulosic substances during extraction [27]. As a result, the obtained fibers showed greater purity and more open structural arrangements. Additionally, the alkaline treatment helped to degrade and eliminate residual proteins, crystalline starches, and lipids [10], contributing to a more favorable fibrous matrix. The looser and more porous microstructure observed in fibers derived from finely milled rice husk significantly enhanced their water- and oil-holding capacities, key functional properties for food applications, particularly in health-oriented products requiring swelling or fat-binding abilities [6]. These findings indicate that optimized particle size enhances the functional properties of rice husk dietary fiber.

#### 3.4.2. FT-IR Spectroscopy

**Figure 4:**
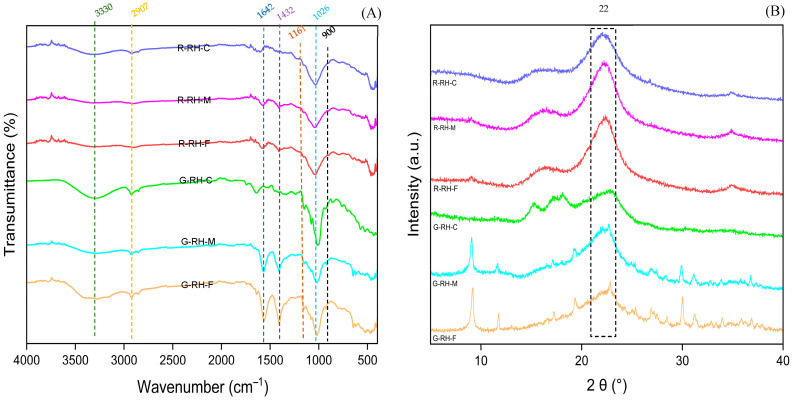
Fourier-transform infrared (FT-IR) spectra (**A**) and X-ray diffraction (XRD) patterns (**B**) of fibers extracted from rice husk fibers. R: fully ripe stage, G: green stage, and RH: rice husk.

FT-IR analysis of dietary fibers from rice husk was performed to identify the functional groups present, as shown in Figure 4A. The obtained spectra reflect the structural components of polysaccharides in both G-RH and R-RH samples. A broad absorption band at 3330 cm^−1^ indicates the presence of hydrogen bonding within the fiber structure, associated with cellulose and hemicellulose [30]. The band at 2907 cm^−1^ corresponds to aliphatic C–H stretching in polysaccharides such as cellulose [38,39,40]. Bands at 1642 and 1161 cm^−1^ are consistent with O–H bending, often related to water absorbed in cellulose fibers [3]. The band at 1432 cm^−1^ is characteristic of C–H bending in the polysaccharide backbone, while bands at 1105 and 1026 cm^−1^ indicate C–O–C glycosidic bond vibrations in cellulose [41]. Additionally, the peak at 900 cm^−1^ is characteristic of β-glycosidic linkages between glucose units in cellulose [42]. These FT-IR results indicate that dietary fibers from both rice husk stages contain cellulose, and the detected functional groups reflect the polysaccharide structures that form the basis of the dietary fiber. Furthermore, the spectral data revealed that particle size influenced the intensity of light absorption. The G-RH sample exhibited stronger absorption bands than R-RH, which may be attributed to additional components such as minerals. These components may exhibit absorption in the 1100–1000 cm^−1^ region (due to Si–O bond vibrations), potentially overlapping with C–O stretching bands (~1020–1040 cm^−1^) of polysaccharides. Additionally, the presence of a peak around 3300 cm^−1^ further indicated O–H stretching, possibly arising from moisture retained in the samples [3]. The increased absorption intensity observed in samples with larger or coarser particle sizes could be attributed to the retention of residual compounds, such as moisture, in higher quantities. These findings show that both the developmental stage and particle size influence the chemical structure of rice husk dietary fiber, which may affect its functional properties.

#### 3.4.3. X-Ray Diffraction

The crystalline characteristics of rice husk dietary fiber were analyzed through X-ray diffraction (XRD). The results demonstrated that variations in growth stages and particle sizes significantly influenced the XRD diffraction patterns (Figure 4B). The box highlights the peak detected in the rice husk dietary fiber. Both G-RH and R-RH samples exhibited distinct diffraction peaks at approximately 2θ ≈ 22.6° and 34.5°, which correspond to the major and minor crystallographic planes of cellulose I. This natural crystalline configuration of cellulose results from the regular arrangement of its polymer chains, maintained by hydrogen bonding and van der Waals forces [17]. Additionally, R-RH showed a sharper peak at 2θ ≈ 22.6° compared to G-RH, indicating a higher degree of crystallinity. This result is consistent with the findings of Yang et al. [43], who observed major peaks for cellulose I at 15.9°, 22.8°, and 33.2° in insoluble dietary fiber from grape pomace. Similarly, Rezvani and Goli [44] reported comparable peaks at 16°, 21°, and 34° in dietary fiber from carrots, confirming the presence of crystalline regions within the cellulose structure. In addition, G-RH-M and G-RH-F displayed a clear low-angle peak (<10°) in the XRD patterns, suggesting crystalline regions of polysaccharides in rice husk dietary fiber, consistent with Cervera et al. [45]. The peak at 30–34° may correspond to a secondary cellulose peak reported by Liu et al. [5]. A small peak near 30° in both samples could also arise from residual inorganic matter, such as minerals or amorphous fiber fractions, as reported in related studies [46,47,48]. The difference in particle size of rice husk dietary fiber affected peak sharpness at 2θ ≈ 22.6°, with finer and medium-ground samples exhibiting higher peak intensity than coarser ones. This could be due to the more efficient extraction of cellulose from smaller particles, leading to a greater presence of crystalline cellulose, thus resulting in more intense diffraction peaks. However, the irregularities observed in some regions of the XRD patterns may be associated with structural changes in cellulose during the extraction process [15]. For example, the extraction process may reduce the crystalline regions or, conversely, remove amorphous regions, allowing cellulose chains to reorganize into more ordered crystalline domains [44]. This study confirmed the presence of cellulose I crystalline structures in rice husk dietary fiber. Such crystallinity may play an important role in enhancing the functional properties of dietary fiber.

### 3.5. Bioactive Compounds and Antioxidant Activities

**Figure 5:**
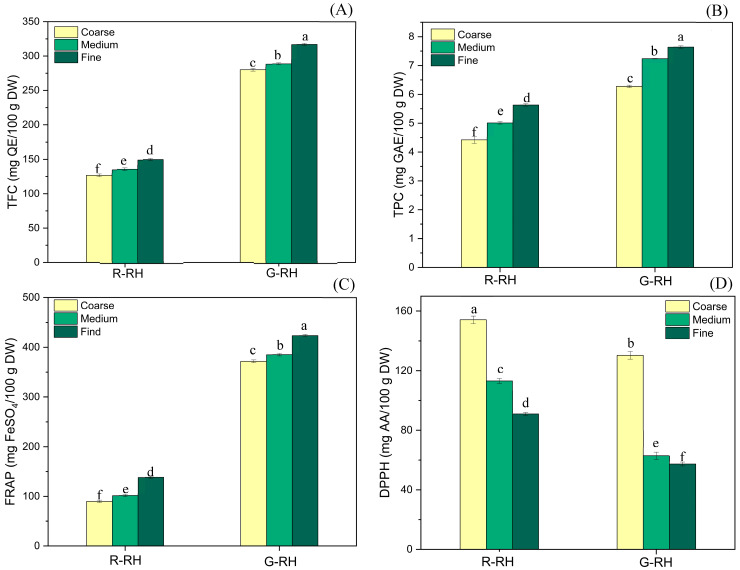
The total flavonoid content (**A**), total phenolic content (**B**), FRAP (**C**), and DPPH (**D**) of fibers extracted from rice husk fibers. Superscripts ^a–f^ indicate that the means represented by the bars are significantly different (*p* < 0.05). R: ripe stage; G: green stage; and RH: rice husk. QE: quercetin equivalents; GAE: gallic acid equivalents, and AA: L-ascorbic acid.

Bioactive compounds are natural substances that exert positive effects on health and physiological functions [14]. Figure 5 illustrates the influence of developmental stages and particle sizes of rice husk-derived dietary fiber on the levels of bioactive compounds and their antioxidant activities. The total flavonoid content (TFC) ranged from 125.75 to 314.94 mg QE/100 g DW (Figure 5A), which is approximately twice the amount reported for wheat bran fiber [12]. Similarly, the total phenolic content (TPC) ranged from 4.42 to 7.66 mg GAE/100 g DW (Figure 5B), which is higher than previously reported values for rice husk DW [4]. The FRAP method was used to evaluate antioxidant capacity, which ranged from 88.01 to 421.99 mg FeSO_4_/100 g DW (Figure 5C), with G-RH samples showing higher values than wheat bran [49]. DPPH radical scavenging activity ranged from 57.61 to 130.43 mg AA/100 g DW (Figure 5D), which is roughly double that reported for soybean hulls [24]. Comparing dietary fibers derived from different developmental stages, G-RH exhibited higher levels of TFC, TPC, and FRAP activity than R-RH, likely due to the naturally higher antioxidant compound content in earlier seed development stages [14]. Moreover, particle size had a significant impact on bioactive compound levels and antioxidant properties. As particle size decreased, TPC, TFC, and FRAP values tended to increase, following the trend fine > medium > coarse. This phenomenon may be attributed to the increased surface area of smaller particles, which enhances reactivity and facilitates the release of antioxidants [12,50]. However, DPPH activity exhibited an opposite trend, decreasing with smaller particle sizes (coarse > medium > fine). This may be attributed to the heterogeneous distribution of antioxidant compounds across different structural components of plant tissue, such as the bran layer and the aleurone layer of rice bran [14]. Moreover, the antioxidant activities measured using the FRAP assay showed a different trend from those measured by the DPPH method, which could be due to the distinct underlying mechanisms of each assay. The DPPH assay evaluates antioxidant capacity based on the ability to donate electrons or hydrogen atoms to neutralize the DPPH radical, whereas the FRAP assay measures electron-donating capacity to reduce Fe^3+^ to Fe^2+^ [51]. These differences suggest that certain bioactive compounds may respond more strongly to one assay than the other, potentially leading to variations in the observed antioxidant activity. Therefore, optimizing particle size during rice husk processing is essential not only for preserving antioxidant potential but also for enhancing the nutritional and functional properties of dietary fiber, particularly in the development of health-oriented functional food products.

### 3.6. Phytosterol

Phytosterols are bioactive compounds that play a significant role in promoting health, particularly in reducing the risk of cancer [52]. These compounds are commonly found in plants such as legumes, seeds, and cereals. The analysis of phytosterol content in dietary fiber extracted from rice husk at different developmental stages Table 4 revealed that both the growth stage and particle size of rice husk-derived dietary fiber influenced the type and quantity of phytosterols. Specifically, R-RH contained only ß-sitosterol. In contrast, G-RH exhibited a greater diversity of phytosterols, including campesterol, stigmasterol, and ß-sitosterol. This finding suggests that rice husk harvested at the green rice husk stage possesses a higher diversity of phytosterols compared to that harvested at the fully ripe rice husk stage. Such differences likely reflect dynamic changes in plant secondary metabolites during seed development. At earlier developmental stages, plants typically synthesize higher levels of bioactive compounds as a defense mechanism against environmental stress [13,14]. As the seed matures, these compounds may undergo transformation or degradation, leading to a natural decline in both the amount and diversity of phytosterols. However, this observation differs from the findings of Marques et al. [1], who reported an absence of detectable phytosterols in mature rice husk compared to maize fiber. Such discrepancies may stem from differences in plant varieties, extraction techniques, or specific developmental stages of the rice samples used. Furthermore, particle size significantly influenced the detectable levels of phytosterols. Finely ground G-RH samples showed higher phytosterol levels compared to medium or coarse particles, indicating that particle size reduction enhances the accessibility and release of bioactive compounds. Smaller particles have a larger surface area for extraction [49], thereby improving extraction efficiency. Nevertheless, phytosterol content can also be affected by the internal structural characteristics of the sample and the sample preparation steps prior to GC-MS analysis, such as hydrolysis, heating, and pressure application. These processes may lead to degradation or isomerization of certain phytosterols, resulting in reduced detectable amounts [52]. Overall, this study highlights that both the developmental stage of rice and the particle size of dietary fiber significantly affect the diversity and quantity of phytosterols in rice husk. These insights are crucial for the development of functional food products aimed at enhancing bioactive compound content.

## 4. Conclusions

This study has provided comprehensive evidence that the physicochemical and functional properties of dietary fiber extracted from rice husk were strongly influenced by both the grain maturity stage and the particle size of the material. R-RH exhibited a higher degree of cellulose crystallinity, while G-RH contained substantially higher levels of β-glucan, total phenolics, flavonoids, antioxidant activity (FRAP), and a more diverse range of phytosterols. These variations are likely due to the natural accumulation of bioactive compounds during early grain development. Particle size also played an important role; smaller particles led to increased yields of cellulose and β-glucan, improved water and oil retention, greater swelling capacity, and higher concentrations of bioactive compounds and phytosterols. These enhancements were supported by SEM images, which showed more porous and expanded structures in smaller particles. The findings demonstrate the potential of rice husk, especially from the green rice husk stage, as a valuable source of functional dietary fiber and bioactive compounds. This research provides meaningful insight for developing nutritious food products and emphasizes the importance of optimizing processing methods to improve the functional and nutritional properties of ingredients derived from rice husk. However, this study focused only on evaluating the properties of dietary fiber from rice husk at two stages (G-RH and R-RH) and primarily analyzed their chemical composition, functional properties, and bioactive compounds at a preliminary level, which may limit the comprehensiveness of the conclusions. Furthermore, all analyses were conducted in vitro, so the physiological effects of these fibers cannot be confirmed with certainty. Future studies should investigate their effects in vivo to validate the potential of rice husk dietary fiber and to support its effective application in the development of functional food products.

## Figures and Tables

**Table 1:** Description of the grain development stage of rice (*Oryza sativa* L.).

Growth Stage	Age (Day After Flowering)	Description
Green rice husk	22–28	Rice husk development begins as the grain matures, which is characterized by the hardening and opacification of the endosperm. Physiological maturity is typically indicated when at least one grain on the main panicle exhibits a brown-colored husk.
Fully ripe rice husk	29–35	Rice husks harvested at the fully ripe stage correspond to grains that have reached their maximum size and hardness. During this stage, the husk color deepens to a darker yellow or brown.

**Table 2:** Physicochemical properties of rice husk fiber.

Group	Sieves	Color			Moisture Content (g/100 g)	a_w_ ^ns^
		L*	a*	b*		
R-RH	Coarse	74.42 ± 0.07 ^b^	−1.65 ± 0.04 ^f^	24.64 ± 0.04 ^e^	3.21 ± 0.01 ^a^	0.21 ± 0.01
	Medium	73.08 ± 0.03 ^c^	−0.73 ± 0.03 ^e^	44.83 ± 0.02 ^b^	3.15 ± 0.01 ^c^	0.21 ± 0.01
	Fine	70.63 ± 0.09 ^d^	1.99 ± 0.04 ^b^	46.27 ± 0.03 ^a^	3.08 ± 0.01 ^e^	0.21 ± 0.01
G-RH	Coarse	78.62 ± 0.06 ^a^	1.35 ± 0.02 ^d^	15.84 ± 0.07 ^f^	3.18 ± 0.01 ^b^	0.21 ± 0.01
	Medium	69.01 ± 0.03 ^e^	1.50 ± 0.02 ^c^	30.45 ± 0.02 ^d^	3.07 ± 0.03 ^d^	0.21 ± 0.01
	Fine	65.19 ± 0.02 ^f^	2.07 ± 0.03 ^a^	30.88 ± 0.01 ^c^	3.04 ± 0.02 ^f^	0.21 ± 0.01

The values are presented as mean ± standard deviation (*n* = 3). Superscripts ^a–f^ indicate that the means in the same column are significantly different (*p* < 0.05). While ^ns^ denotes no significant difference (*p* > 0.05). R: ripe stage, G: green stage, and RH: rice husk.

**Table 3:** Water-holding capacity (WHC), oil-holding capacity (OHC), and swelling capacity (SC) of rice husk fiber.

Group	Sieves	Water-Holding Capacity (g/g)	Oil-Holding Capacity (g/g)	Swelling Water Capacity (mL/g)
R-RH	Coarse	1.44 ± 0.01 ^e^	1.51 ± 0.03 ^g^	0.35 ± 0.03 ^g^
	Medium	5.31 ± 0.02 ^b^	2.35 ± 0.02 ^d^	0.61 ± 0.02 ^f^
	Fine	5.52 ± 0.03 ^a^	2.47 ± 0.17 ^c^	0.74 ± 0.02 ^d^
G-RH	Coarse	1.24 ± 0.03 ^f^	1.62 ± 0.01 ^f^	0.88 ± 0.01 ^c^
	Medium	4.55 ± 0.02 ^d^	3.17 ± 0.03 ^b^	1.10 ± 0.02 ^b^
	Fine	5.25 ± 0.02 ^c^	3.44 ± 0.02 ^a^	1.21 ± 0.01 ^a^

The values are presented as mean ± standard deviation (*n* = 3). Superscripts ^a–g^ indicate that the means in the same column are significantly different (*p* < 0.05). R: ripe stage, G: green stage, and RH: rice husk.

**Table 4:** Chemical composition, β-glucan, and phytosterol of rice husk fiber.

Sample	Sieves	Cellulose (g/100 g DW)	Moisture (g/100 g DW)	Ash (g/100 g DW)	Protein (g/100 g DW)	Lipid (g/100 g DW)	β-Glucan (mg/100 g DW)	Campesterol (µg/g DW)	Stigmasterol (µg/g DW)	β-Sitosterol (µg/g DW)
R-RH	Coarse	41.27 ± 0.13 ^e^	3.27 ± 0.02 ^b^	3.54 ± 0.02 ^f^	4.33 ± 0.02 ^b^	8.76 ± 0.02 ^a^	66.37 ± 0.06 ^e^	BDL	BDL	28.98 ± 0.07 ^e^
	Medium	76.30 ± 0.35 ^b^	1.86 ± 0.01 ^e^	5.21 ± 0.02 ^e^	2.07 ± 0.02 ^d^	6.35 ± 0.03 ^c^	72.78 ± 0.23 ^d^	BDL	BDL	72.33 ± 0.05 ^d^
	Fine	82.04 ± 0.13 ^a^	1.55 ± 0.02 ^f^	6.25 ± 0.03 ^c^	1.79 ± 0.01 ^e^	4.96 ± 0.01 ^e^	85.14 ± 0.97 ^b^	BDL	BDL	94.68 ± 3.74 ^c^
G-RH	Coarse	37.49 ± 0.24 ^f^	4.97 ± 0.01 ^a^	5.96 ± 0.02 ^d^	5.01 ± 0.01 ^a^	7.94 ± 0.03 ^b^	67.51 ± 1.72 ^e^	BDL	BDL	BDL
	Medium	68.02 ± 0.04 ^d^	3.11 ± 0.02 ^c^	7.90 ± 0.03 ^b^	2.73 ± 0.24 ^c^	5.31 ± 0.02 ^d^	80.02 ± 1.83 ^c^	BDL	326.09 ± 1.41 ^b^	719.46 ± 1.57 ^b^
	Fine	73.16 ± 0.15 ^c^	3.02 ± 0.01 ^d^	9.15 ± 0.02 ^a^	2.58 ± 0.23 ^c^	4.95 ± 0.03 ^e^	92.85 ± 2.36 ^a^	293.25 ± 4.82 ^a^	375.13 ± 3.98 ^a^	734.45 ± 4.95 ^a^

The values are presented as mean ± standard deviation (*n* = 3). Superscripts ^a–f^ indicate that the means in the same column are significantly different (*p* < 0.05). R: ripe stage, G: green stage, and RH: rice husk. BDL; below the detection limit BDL.

## Data Availability

The original contributions presented in this study are included in this article. Further inquiries can be directed to the corresponding author.
